# Sex and Electrode Configuration in Transcranial Electrical Stimulation

**DOI:** 10.3389/fpsyt.2017.00147

**Published:** 2017-08-14

**Authors:** Michael J. Russell, Theodore A. Goodman, Joseph M. Visse, Laurel Beckett, Naomi Saito, Bruce G. Lyeth, Gregg H. Recanzone

**Affiliations:** ^1^Aaken Laboratories, Davis, CA, United States; ^2^Sutter Center for Psychiatry, Sacramento, CA, United States; ^3^Division of Biostatistics, Davis School of Medicine, University of California, Davis, Davis, CA, United States; ^4^Department of Neurological Surgery, University of California, Davis, Davis, CA, United States; ^5^Center for Neuroscience, University of California, Davis, Davis, CA, United States; ^6^Department of Neurobiology, Physiology and Behavior, University of California, Davis, Davis, CA, United States

**Keywords:** transcranial electrical stimulation, high density stimulation, direct current stimulation, alternating current stimulation, MRI modeling, electrical targeting, 10–20 system

## Abstract

Transcranial electrical stimulation (tES) can be an effective non-invasive neuromodulation procedure. Unfortunately, the considerable variation in reported treatment outcomes, both within and between studies, has made the procedure unreliable for many applications. To determine if individual differences in cranium morphology and tissue conductivity can account for some of this variation, the electrical density at two cortical locations (temporal and frontal) directly under scalp electrodes was modeled using a validated MRI modeling procedure in 23 subjects (12 males and 11 females). Three different electrode configurations (non-cephalic, bi-cranial, and ring) commonly used in tES were modeled at three current intensities (0.5, 1.0, and 2.0 mA). The aims were to assess the effects of configuration and current intensity on relative current received at a cortical brain target directly under the stimulating electrode and to characterize individual variation. The different electrode configurations resulted in up to a ninefold difference in mean current densities delivered to the brains. The ring configuration delivered the least current and the non-cephalic the most. Female subjects showed much less current to the brain than male subjects. Individual differences in the current received and differences in electrode configurations may account for significant variability in current delivered and, thus, potentially a significant portion of reported variation in clinical outcomes at two commonly targeted regions of the brain.

## Introduction

Transcranial electrical stimulation (tES) is among the oldest procedures in neurology, with the earliest reports going back to the treatment of headaches and melancholia with electric fish in the first century CE (Common Era) (1). Since modern electronics have replaced electric fish as a mean of stimulation, transcranial stimulation has been widely applied to test experimental and clinical conditions of motor (2–5), psychiatric (6–8), and cognitive (9–12) processes. Stimulation with relatively high current levels designed to initiate motor-evoked potentials and induce seizures has proven to be a reliable technique (13–17).

Low intensity alternating current (AC) and direct current (DC) stimulations have become the focus of non-invasive interventions and neuromodulation in many laboratories and several commercial companies. Their work has raised interest in more effective targeting of specific brain regions to potentially improve these techniques to maximize effectiveness and minimize risks and side effects to the patients.

In spite of a 2,000-year-old history of electrical stimulation of the brain, it has not been possible to accurately determine how much current reaches the brain given different stimulation parameters. Estimates of current delivered to the brain have been roughly based on the intensity of the stimulation at the electrodes. Knowledge of the dosage of current that a subject receives is a critical factor for both research and clinical practice. Recent meta-analyses have shown that there is a great deal of variation in the effects of electrical stimulation on outcome measures in research studies, but these analyses have not accounted for the differences in electrode configurations and current dosage applied to the subjects (2, 11, 18–22).

The recognition that there are individual differences in the current received by a particular subject or patient has been the focus of multiple laboratories (23–26). The inconsistencies between individuals are due in part to the variation in the specific electrical resistance of tissues such as skin, muscle, bone, gray matter, white matter, and cerebral spinal fluid and the differences in the morphometry of individual heads. One type of individualized MRI modeling divides the MRI image of the cranium into different regions (bone, spinal fluid, white matter, etc.) and assigns normative values to each segment based on values taken from the literature in order to create a realistic looking composite model. This segmented approach has been used by many laboratories—including this one (27). However, segmentation has some limitations. For example, segmentation assumes that there is only one type of tissue within a segment, ignores the range of resistivity that may exist within a tissue type, and overlooks individual differences in tissue resistivity. We have recently adopted an alternate MRI assessment method that avoids these segmentation issues and calculates conductivity by determining the amount of conductive salt water within each voxel of an imaged tissue (28). This modeling method has been shown to correlate very strongly with the current measured at the scalp (*r* = 0.93) and demonstrated important individual differences in the amount of current received by the brain when the traditional 10–20 system was used to determine electrode placement (28). Individual skull differences are particularly important because of the high variability in skull density across individuals and the substantial changes in skull density over the lifespan (29, 30).

**Figure 1:**
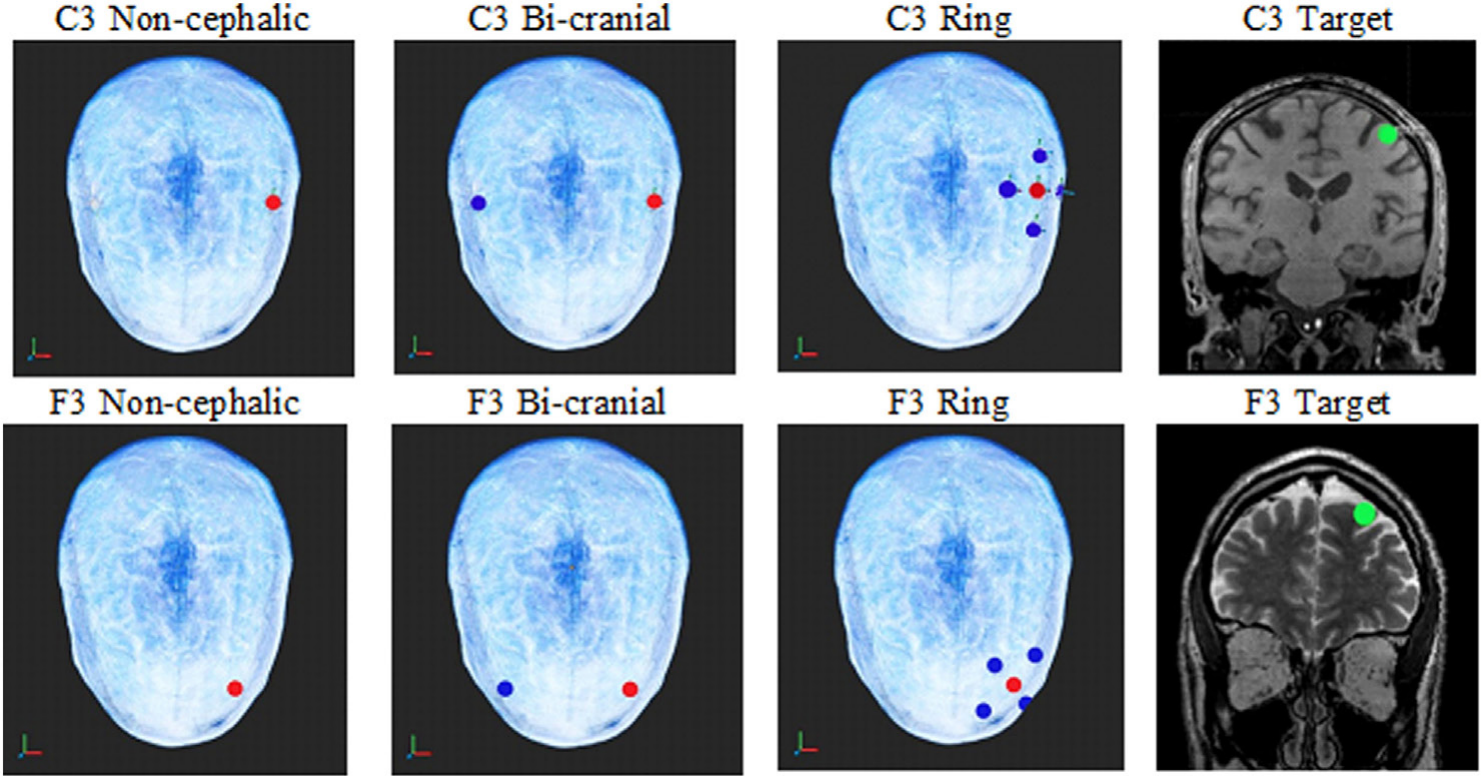
The top row shows the electrode configurations on the top of the head for non-cephalic, bi-cranial, and ring. The green circle in the coronal MRIs shows an image of the current sampling area (green dot) under the C3 and F3 electrodes where the current calculations were made for each configuration. Red electrodes are positive, and blue electrodes are negative. The top row is the C3 electrode locations, and the bottom is the F3 locations.

Three different types of electrode configurations make up the bulk of both research and clinical treatments. Bi-cranial stimulation is the most common configuration for cortical activation. It has become the standard of care for several procedures including intraoperative monitoring (14, 15, 31), and it is used extensively for low intensity DC and AC stimulations in research and clinical treatment. In this configuration, electrodes are placed on opposite sides of the scalp. The ring configuration (sometimes called high density) is a relatively new technique (32) and was introduced as an alternate to the large sponge electrodes still in use by some laboratories. In the ring configuration, 1 cm electrodes are placed in a circle around a central electrode of the opposite polarity (Figure 1), with the idea that the currents will not be spread across the scalp and will therefore stimulate the underlying cortex in a smaller area (32). The ring configuration has become common for low current DC (33–42) and AC (33) stimulations. Non-cephalic stimulation is the least common configuration, but has been used effectively (43). In this configuration, one electrode is placed on the skull and the second electrode of opposite polarity is placed on the torso or shoulders. The questions posed in the present study are how effective these three electrode placements are in delivering electrical current to the brain and how individual variability between subjects influences current delivery.

## Materials and Methods

### Subjects

This study protocol was approved by the Sutter Institute for Medical Research Institutional Review Board, and participating individuals gave both written consent and verbal informed consent. Consent was assured and recorded by having each subject sign an informed consent form after the protocol was discussed and each subject’s questions were fully answered. Twelve males and eleven females were enlisted in the study. The age ranges of male and female patients were 28–68 and 21–62 years, respectively, and none had known anatomical anomalies. They were recruited from the patients and staff of the Sutter Center for Psychiatry.

### MRI Procedures

After receiving each subject’s approval, MRIs were collected according to the Aaken Insite Protocol (44). Briefly, a combination of MRIs [T1, T2, and proton density (PD)] was recorded. T1, T2, and PD imaging each capture different aspects of the water (hydrogen) present in tissues, but each alone fails to provide a precise measure of brain conductivity. Consequently, for each subject, the data from the three types of MRI were combined to model conductivity. Diffusion weighted recordings were not used in this instance because they are considered to be unreliable for the cerebral cortex (45). The recordings were obtained on a 3 T General Electric MRI (Discovery M750) machine with 1 mm × 1 mm × 1 mm slice spacing.

### Virtual Electrodes

After the head models were formed from the combined MRIs, virtual stimulating electrodes were placed on each modeled scalp to represent ring (high density), bi-cranial, and non-cephalic referencing based on the 10–20 electrode configuration system. The exact electrode placements were determined by a computer algorithm. The target current densities were measured at a 10 mm diameter sphere directly under the C3 and F3 locations and were in identical locations for each of the three stimulation electrode configurations. For the C3 non-cephalic electrode configuration, the positive electrode was balanced by an electrode of equal negativity placed below the neck. For the bi-cranial electrode configuration, the positive C3 had a negative C4 electrode of equal current on the opposite side of the head. For the C3-ring electrode configuration, the positive C3 had four surrounding negative electrodes at one quarter the value of the C3 electrode and 3 cm distant (see Figure 1).

A second scalp location used the frontal lobe-positive electrode location of F3 to non-cephalic below the neck negative reference. For the bi-cranial condition, the negative virtual electrode was placed at F4. For the ring condition, four negative electrodes at one quarter the value of the F3 were placed on the scalp 3 cm distant to the positive electrode.

The current density was calculated within a 1 cm sphere of brain tissue directly under the anodal (+) C3 and F3 electrodes, respectively, at the exact same scalp locations for each subject and condition.

### Modeling Procedures

The modeling procedures were based on a previously published protocol (28). This procedure mapped the conductivity of cranial tissue, a three-dimensional (3D) measurement of the hydrogen distribution in head and brain is needed rather than the typical individual slice MRI record. To achieve this, data from the T1, T2, and PD MRIs were combined into a single 3D representation of the amount of water within a tissue and a conversion equation was applied to achieve an index of resistivity, yielding the individual subject’s resistivity model. The resistivity to the MRI intensity is expressed by the formula:

(1) $$R(v)=K{\left(1-v\right)}^{E}+D$$

where *v* ∈ [0,1] is the normalized intensity of the combined image at the given voxel; *R* is the resistivity; and *K* = 16,000, *E* = 4, and *D* = 65 are the adjustable parameters.

### Finite Element Analysis

Finite element and boundary conditions are given in detail at Russell et al. (28). Briefly, the subject’s resistivity distribution was translated to a rectangular prismatic linear finite element model. The model matrix equation and boundary conditions were formulated from the Galerkin equations (46). Solutions to the system matrix equations were obtained by using the conjugate gradient method (47). The finite element models solved the Laplace equation, and current densities within the models were determined from the finite element model solution by calculating current densities for each voxel.

### Modeling Current Density

Current density estimates were achieved by placing virtual electrodes (red [+] and blue [−] in Figure 1) according to the 10–20 system for electrode placement, and current intensities were applied at 0.5, 1.0, or 2.0 mA. The sampling area for current density was a 10 mm diameter virtual sphere within the brain (green circle in Figure 1 coronal view) directly under either the C3 electrode or the F3 electrode. The sphere defined the voxels in the cortical region assessed for the analysis. The outcome variable was the mean current level for the 1 mm voxels within the 10 mm diameter sphere (~526 mm).

Three different stimulated current intensities (0.5, 1.0, and 2.0 mA) were used to determine the current density at the target electrode locations. To ensure consistency, the positive C3 or F3 electrode was not moved, but the negative references were adjusted for each configuration.

### Data Analysis

The data were analyzed using R (version 3.2.2). Current was measured at two locations, three configurations, and three intensities, thus producing 18 measurements per subject. Quantile–quantile (*Q*–*Q*) plots of current density showed non-normality of the residuals. We examined both log and square root transformations and found that a log transformation was most effective in correcting the upper tail non-normality, although it introduced a smaller amount of lower tail non-normality. We report the log-transformed results as our primary outcome evaluation since this avoided excess influence of higher values.

We performed a repeated measures analysis of variance (ANOVA) mixed effects analysis with random effect of subject. We tested whether the log-transformed current density (microampere per centimeter square) (the dependent variable) was affected by the four independent variables: electrode configuration (non-cephalic, bi-cranial, or ring), stimulation intensity (0.5, 1.0, or 2.0 mA), subject’s sex (male or female), or stimulation site (C3 or F3). Next, we added two-way interactions to determine whether the effects of configuration were modified by intensity, location, or sex; whether effects of intensity were modified by location or sex; and whether effects of location were different for males and females. We chose as reference values for comparison the ring configuration, 0.5 mA intensity, temporal location, and female sex.

## Results

**Figure 2:**
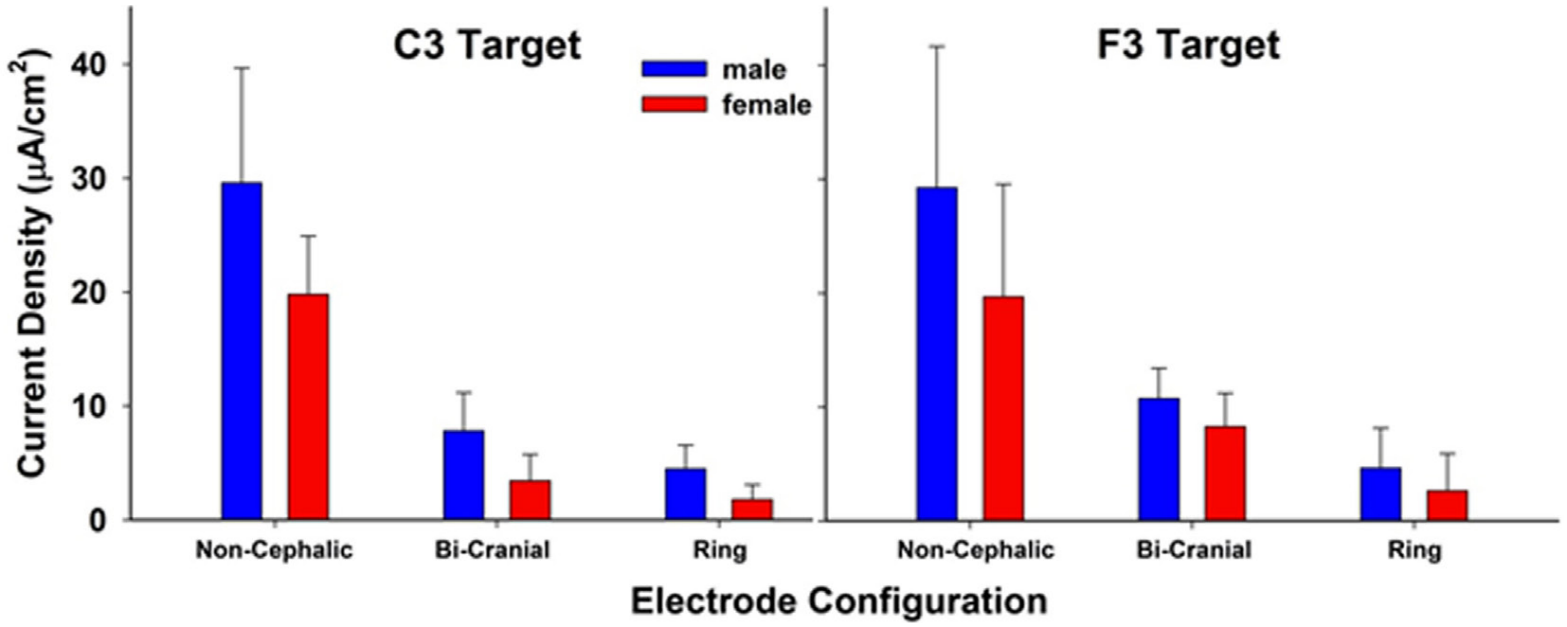
Modeled current density in a 10 mm diameter sphere within the brain parenchyma directly under the stimulating scalp electrode at sites C3 and F3. Each bar is the resulting average current density of three stimulation current values (0.5, 1.0, and 2.0 mA). There were significant effects of electrode configuration and sex. The values between stimulation sites C3 and F3 were not significantly different. The bars represent mean current density and the whiskers represent the SDs.

Table 1 shows means and SDs (prior to log transformation) for current densities (microampere per centimeter square) for each of the 18 combinations of configuration, location, and current intensity, overall and separately for males and females. The increase in measured density with current intensity is readily apparent across all conditions (see Figure 2), but patterns of heterogeneity of variances and long tails of the distributions within cells necessitated log transformation before further analysis.

**Table 1:** Current density (μA/cm^2^) was modeled in a 10 mm diameter sphere within the brain parenchyma directly under the stimulating scalp electrode at sites C3 and F3.

	C3 non-cephalic			C3 bi-cranial			C3-ring		
Stimulation (mA)	0.5	1.0	2.0	0.5	1.0	2.0	0.5	1.0	2.0
Female mean (SD)	7.94 (2.80)	16.71 (4.65)	34.79 (9.51)	1.42 (1.03)	3.02 (1.90)	5.9 (3.95)	0.83 (0.63)	1.66 (1.27)	2.9 (2.25)
Male mean (SD)	13.36 (4.59)	25.2 (8.78)	50.3 (17.55)	3.43 (1.40)	6.87 (2.80)	13.17 (5.98)	1.94 (0.91)	3.83 (1.78)	7.66 (3.60)
Overall mean (SD)	10.77 (4.66)	21.11 (8.18)	42.88 (16.06)	2.47 (1.58)	5.03 (3.07)	9.69 (6.22)	1.41 (0.96)	2.79 (1.88)	5.38 (3.83)

	**F3-non-cephalic**			**F3 bi-cranial**			**F3-ring**		

Stimulation (mA)	0.5	1.0	2.0	0.5	1.0	2.0	0.5	1.0	2.0
Female mean (SD)	8.42 (4.23)	16.83 (8.44)	33.75 (17.01)	3.43 (1.29)	6.87 (2.58)	14.51 (5.14)	1.14 (1.39)	2.26 (2.79)	4.54 (5.57)
Male mean (SD)	12.60 (5.23)	25.12 (10.65)	50.07 (21.27)	4.59 (1.14)	9.11 (2.18)	18.52 (4.71)	2.03 (1.56)	3.96 (3.07)	7.89 (5.92)
Overall mean (SD)	10.60 (5.14)	21.16 (10.34)	42.26 (20.67)	4.03 (1.33)	8.04 (2.59)	16.60 (5.23)	1.60 (1.52)	3.15 (3.00)	6.29 (5.88)

*C3 is above the temporal portion of the parietal bone, and F3 is above the frontal bone. The values in the table are mean values for modeled current density at stimulation site targets (C3 or F3) with stimulation currents of 0.5, 1.0, and 2.0 mA, separately for males and females and overall*.

Analysis of variance of log-transformed data, examining the overall effects of configuration, sex, location measured, and intensity of current, showed substantial differences in mean current density between males and females, across all experimental conditions (Table 2). On average, males had nearly double the current intensity reported compared to females (95% CI 1.36- to 2.72-fold, *p* < 0.01). The ring configuration had the lowest density regardless of sex of participant, location measured, or current intensity, with the bi-cranial configuration yielding 2.8-fold higher current density and non-cephalic 9.7-fold higher (both *p* < 0.01). The F3 location had 17% greater current density measured. As expected, for every doubling of current intensity, the measured current density doubled (*p* < 0.01).

**Table 2:** Analysis of variance results: main effects of fixed factors (configuration, sex, location, and intensity of current) on log (current density).

Effect		Coefficient	95% CI	*p*-Value	Exp(coef)
(Intercept)		−0.44			
Configuration	Ring	0^a^			
	Bi-cranial	1.03	(0.90, 1.16)	<0.01	2.807
	Non-cephalic	2.27	(2.14, 2.40)	<0.01	9.700
Sex	Female	0^a^			
	Male	0.66	(0.31, 1.00)	<0.01	1.928
Location	C3	0^a^			
	F3	0.16	(0.05, 0.27)	<0.01	1.173
Intensity	0.5 mA	0^a^			
	1 mA	0.70	(0.57, 0.83)	<0.01	2.012
	2 mA	1.39	(1.25, 1.52)	<0.01	3.995

*^a^Reference group*.

Table 3 shows the ANOVA results adding additional tests for possible effect modification (interactions), which found that significant two-way interactions related to configuration, location, and sex males had nearly a threefold greater current density than females in the reference condition of ring configuration and location C3 (male coefficient in Table 3). The effect of sex was significantly lower, however, in other settings (27% lower in the bi-cranial than in ring, 39% lower in non-cephalic than in ring, and 22% lower in F3 than in C3, from interaction terms.) Thus, males had greater current density than females regardless of configuration or location of measurement, but the difference was greatest for the ring configuration and C3 location. Table 3 expands on the summary of Table 2, which shows only the estimated average effect across all combinations of settings. Location, interestingly, demonstrated no effect for either sex in the reference condition of ring configuration (main effect for F3, *p* = 0.97) and the non-cephalic configuration did not differ from the ring configuration in this regard (*p* = 0.57), but there was a significant 2.25-fold increase in current density for F3 compared to C3, which was found only in the bi-cranial configuration. Current density continued to show a straightforward twofold increase for each doubling of current intensity, regardless of other factors, and current intensity did not otherwise modify the effects of configuration, location, or sex (results not shown). There were no significant three-way interactions.

**Table 3:** Analysis of variance results: interaction effects of fixed factors (main effects plus interaction of configuration and location, configuration and sex, location and sex) on log(current density).

Effect		Coefficient	95% CI	*p*-Value	Exp(coef)
(Intercept)		−0.5			
Configuration	Ring	0^a^			
	Bi-cranial	0.79	(0.58, 1.00)	<0.01	2.20
	Non-cephalic	2.49	(2.28, 2.70)	<0.01	12.06
Sex	Female	0^a^			
	Male	1.05	(0.66, 1.44)	<0.01	2.86
Location	C3	0^a^			
	F3	−0.004	(−0.20, 0.19)	0.97	1.00
Intensity	0.5 mA	0^a^			
	1 mA	0.70	(0.58, 0.82)	<0.01	2.01
	2 mA	1.39	(1.27, 1.50)	<0.01	4.00
**Interactions**					
Configuration × loc	Bi-cranial × F3	0.81	(0.57, 1.05)	<0.01	2.25
	Non-ceph × F3	0.07	(−0.17, 0.31)	0.57	1.07
Configuration × sex	Bi-cranial × male	−0.32	(−0.56, −0.08)	0.01	0.73
	Non-ceph × male	−0.49	(−0.73, −0.25)	<0.01	0.61
Location × sex	F3 × male	−0.25	(−0.44, −0.05)	0.02	0.78

*^a^Reference group*.

A second finding of note was that there was a large variability of current densities across subjects tested in each configuration. For example, with the 2.0 mA stimulation, the current density ranged between 10 and 89 µA/cm^2^ (an 8.9-fold difference) in the non-cephalic configuration, 1.02 and 14.37 µA/cm^2^ (a 14-fold difference) in the ring configuration, and 1.48 and 23.77 µA/cm^2^ (a 16-fold difference) in the bi-cranial configuration. The 1 and 0.5 mA stimulations had similar degrees of variation between same-sex subjects.

**Figure 3:**
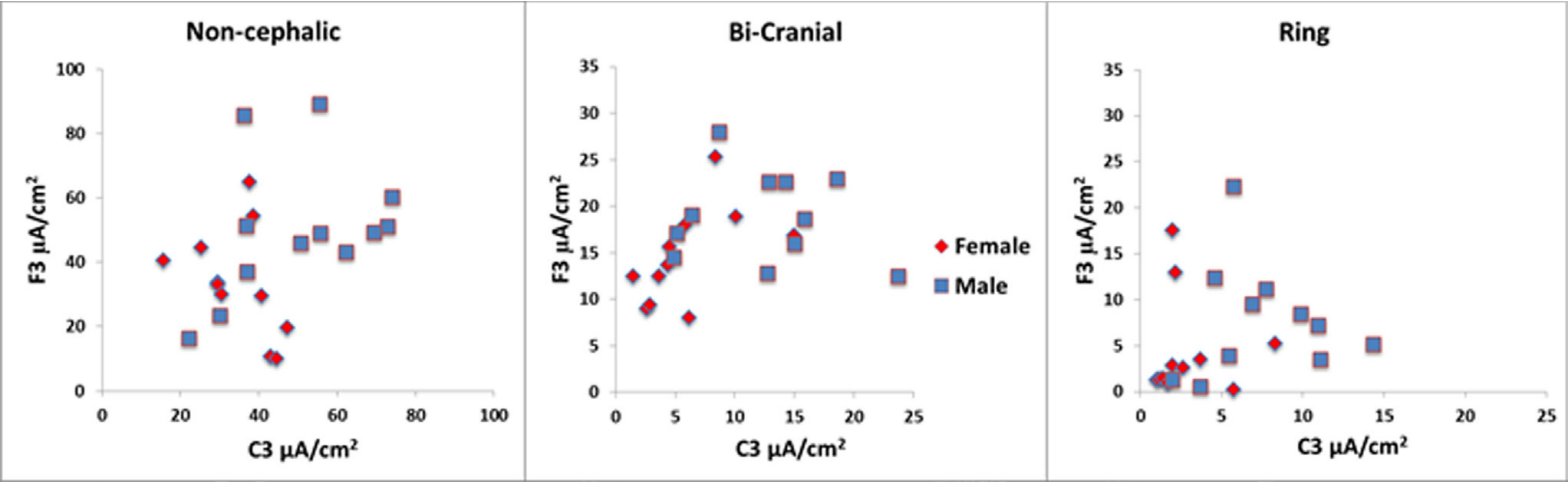
The scatter plots above show wide range of values within each configuration for the 2 mA stimulation. In the ring condition, some values were less than 1 µA/cm^2^. Note that the vertical and horizontal axes are different for each of the configurations. The red symbols are females, and the blue symbols are males.

**Figure 4:**
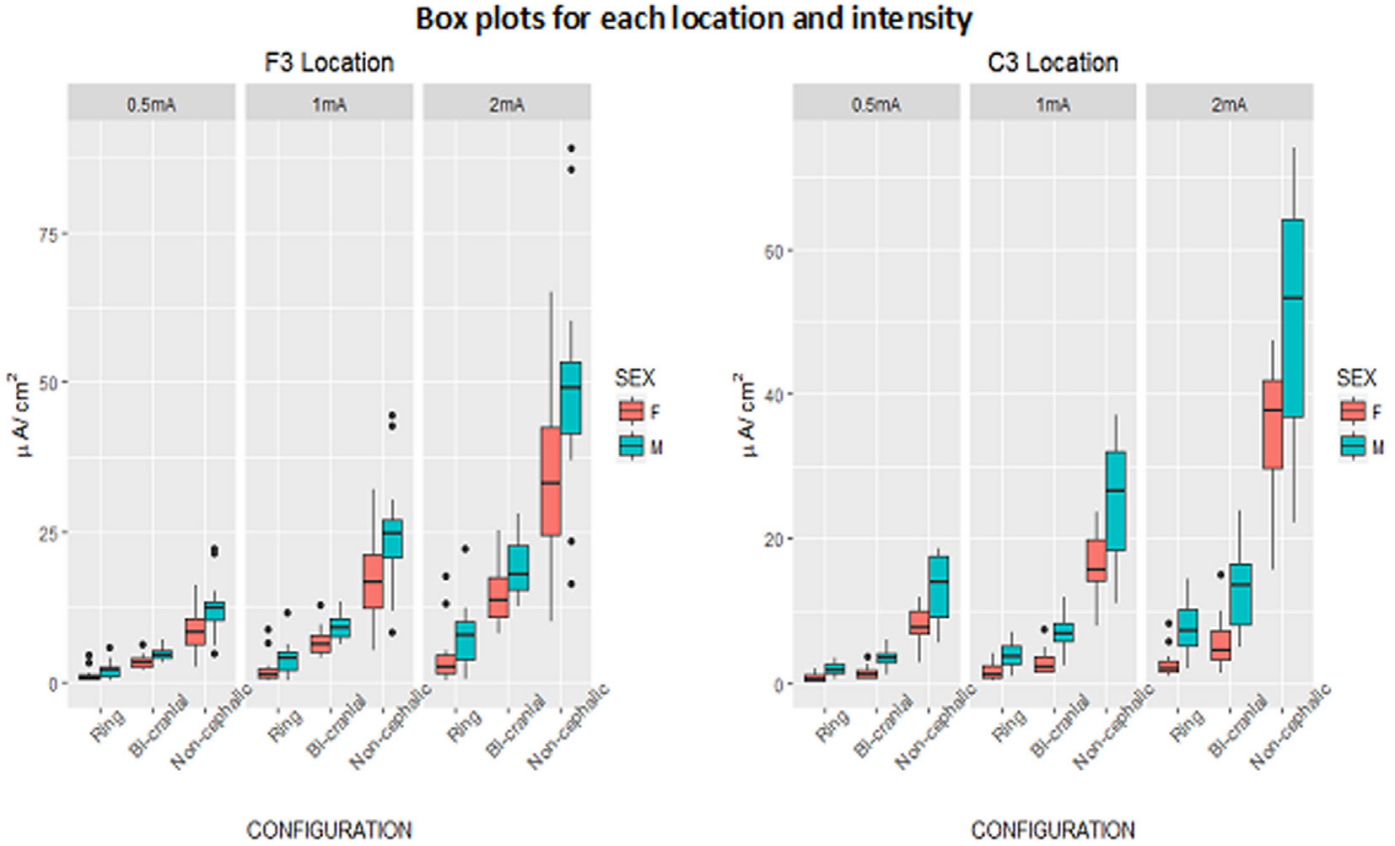
Box plots of log-transformed current density (microampere per centimeter square) by configuration and current intensity, showing males and females, separately for F3 and C3 locations. Box denotes middle 50% of people, bar across box denotes median, whiskers indicate range of observations within 1.5 box-lengths of upper and lower ends of box, and circles denote outliers beyond 1.5 box-lengths.

Given this individual variability, we also sought to determine if individual subjects showed similar current densities across different electrode conditions, for example, if they had a particular skull or scalp morphology that was consistent across the cranium. If this were the case, then one would expect a strong correlation between the current densities measured at the C3 and F3 electrodes within individuals. The scatter plots in Figure 3 address this issue and show very weak correlations (R range: 0.1–0.3), indicating high variability across scalp locations even within a single subject. Thus, measurements at one location in the brain are minimally correlated with that of a different location when using the same electrode configuration and stimulus intensity. Figure 4 is a visual representation of the log-transformed current density illustrating the male and female differences and the range current densities in µA/cm^2^.

## Discussion

This report used MRI-based tissue resistivity modeling to compare three typically used tES electrode configurations in male and female subjects at two scalp locations on current density delivered to the brain. We found that there is a large effect of electrode configuration and sex on the current densities modeled under the electrode, as well as a great deal of individual variability that was not consistent across the different electrode configurations. These results indicate that very different stimulation protocols could be necessary between different individuals in order to effectively stimulate the brain in a consistent manner.

### Electrode Configuration

There were large differences in the currents received at the target depending on both the simulated current intensity (0.5, 1.0, or 2.0 mA) and the electrode configuration. Of the three electrode configurations tested, the ring configuration showed the lowest current density at both the C3 and F3 electrode sites compared to the other two configurations. This is surprising since the ring configuration was previously reasoned to better focus the electrical currents to the desired location (32, 48). At the lowest stimulation intensities, some subjects received less than 1.0 µA of current. The non-cephalic electrode configuration, which is least often used, showed the highest current density under both the C3 and F3 electrodes at all three intensities tested. The non-cephalic configuration delivered 6–9 times the current density compared to the ring configuration and 2.5–4.4 times the current density compared to the bi-cranial configuration. Thus, the non-cephalic condition was the most efficient at delivering current to the defined target area of brain with the least amount of current passing through the scalp tissues where somatosensory and pain receptors are predominant. The 0.5 mA stimulation level with the non-cephalic condition delivered a higher mean level of current to the brain than the 2 mA stimulation level in the ring configuration. This suggests that at least some of the variability seen in published reports can be attributed to the type of electrode configuration used.

Some of the effects observed in published reports with bi-cranial and ring configurations may be due to stimulation of the cranial nerves rather than stimulation of the cerebral cortex. Trigeminal stimulation has been reported to effectively treat psychiatric conditions and depression (49). Because more current goes through the sensory receptors within the scalp for both the ring and bi-cephalic conditions, there is a higher likelihood that a portion of the reported effects seen in published reports may be due to placebo effects resulting from more intense scalp sensations. At the lowest levels of stimulation, the ring configuration is likely to be the least effective for applications requiring a minimum of brain stimulation. If very low current levels to the brain are needed, it may be more desirable to use a lower level of non-cephalic stimulation and have less current passing laterally across the surface of the scalp. Of note from the data shown in Figures 2 and 3 is the large variability in the current densities modeled across individuals. This is consistent with the notion that individual variability in cranial morphology could be a primary determinant of the amount of current penetrates neural tissue and may underlie much of the wide variability in the results both within and between clinical and research studies.

### Sex Differences

Across different electrode configurations and stimulus intensities, female subjects received less current to the brain compared to males, with the difference most prominent in the bi-cranial and ring conditions and at the C3 temporal location compared to the F3 or frontal location. Women also received more current across the scalp and likely experience a higher level of discomfort proportional to the amount of current passing across the scalp sensory receptors. The sex difference is consistent with a previous report that showed no difference in cranial thickness or distance from the stimulating electrode but identified differences due to an increase in cranial density among females and more porous cancellous bone in males (26).

The sex differences are greater in the C3 temporal location than in the F3 frontal location and appear to reflect the higher density of the frontal bone. The sex differences observed at C3 would not be evidenced if a simple segmentation method of modeling was applied that assumed all bone to be of the same density or configuration in all locations. Furthermore, the sex differences noted here would likely change over additional regions of the skull, where sex-based differences in bone density are larger or smaller than under the C3 location. Indeed, bone density also varies with age, race, and medical history and these factors may also influence the current received when the brain is stimulated transcranially. These differences should also be considered as additional sources of variability seen across subjects and studies using tES.

Targeting and dosing of tES has shown little more than incremental improvements since the adoption of the 10–20 system (that was originally designed for recording electroencephalograms) in 1958 (50). MRI modeling may provide an opportunity to significantly reduce one source of variance and improve the technique.

### Limitations

This study shows the relative current received under different electrode configurations, but does little to determine the optimal amount of current needed to achieve stimulation at a particular current intensity. Presumably, there is an ideal current dosage that is necessary to achieve effective neuromodulation.

This study modeled the mean amount of current within all of the voxels within the target sphere but ignored the variance between voxels within the sphere. Differences in conductivity relating to blood vessels, connective tissue, cerebral spinal fluid, etc., could influence the modeled results. This is a concern as the target sphere was uniformly placed in each subject by the 10–20 system and not by the individual’s topography. Each subject would not have equivalent amounts or types of tissue within the target sphere; position and topography of the various structures is different for each individual. However, this study was designed to determine how much variability does exist when using the standard 10–20 system and these results underscore the value in individualizing electrode placements to effectively target electrical current to specific brain regions. The study models assumed a perfect connection between each electrode and the skin surface and did not attempt to account for imperfect connections due to variations in skin, hair, electrode paste, electrode material, electrode shape, and other factors that would likely further increase variability. Also individual differences in current distributions within a single electrode configuration are not addressed in this study but are shown to vary greatly in an earlier publication (28).

Additional metrics could be added to the model to improve accuracy. For example, the range of distributions and current directions that occur when individual gyri and sulci are represented for each subject would be different and would influence the response of neural tissue to stimulation. In addition to current density, it would be important to know the differences in the tangential and radial components of the electric field, but since this geometry is different for each individual brain and poorly delineated at the cortex with MRI tractography, we have not attempted to define directional components of the current field in this study.

One electrode type was modeled, but many other electrode types are in use including sponges, needles, and concentric, where each has different properties that affect current flow and distribution. Thus, similar modeling of those electrode configurations will likely produce results with a great deal of individual variability and difficulty in predicting the amount of current under a given electrode in the absence of such modeling.

Finally, this study only examined two locations as an illustration of the broader problem of individual variation in electrode configurations. The skull is a high resistance and anatomically complex structure with multiple bone thicknesses and densities that impact current flow at different regions of the head. Similarly, the scalp includes multiple tissues and thicknesses in different regions. An analysis of other regions over the frontal and parietal regions of the skull would likely produce different results.

## Conclusion

Current dosage and targeting are determinate issues in tES. Electrode configuration is an important factor along with stimulus intensity in defining the amount of current received by the brain. This study compared three electrode configurations. The data indicated that the ring configuration closely followed by bi-cranial stimulation delivered the least current to the brain, while the non-cephalic configuration delivered the most current. The female subjects received significantly less current to the brain than the male subjects. Skull and tissue differences are inherent in the subjects and are not readily changed, but the configurations of the electrodes are easily modified and should be considered in light of these findings.

## Ethics Statement

This study protocol was approved by the Sutter Institute for Medical Research Institutional Review Board, and participating individuals gave both written consent and verbal informed consent. Consent was assured and recorded by having each subject sign an informed consent form after the protocol was discussed and each subject’s questions were fully answered in accordance with the Declaration of Helsinki.

## Author Contributions

MR did the experimental design. TG did the design and funding, JV was the text editor and helped with the concept. LB did the data analysis and interpretation, NS did data investigation and editing, BL helped with the study design and data analysis, GR was the senior researcher and did data interpretation.

## Conflict of Interest Statement

Aaken Laboratories is a holding company that maintains the intellectual property rights for the software used in this study. MR, TG, and JV each have shares in Aaken Insite, a service company that uses MRI modeling described in this study. This does not alter our adherence to policies on sharing data and materials. BL, GR, LB, and NS have no conflicts.
